# Comparison of the efficacy of subxiphoid and intercostal uniportal video-assisted thoracoscopic surgery in patients with early-stage non-small cell lung cancer

**DOI:** 10.12669/pjms.41.2.11096

**Published:** 2025-02

**Authors:** Zhiqiang Fu, Liguo Wang, Weigao Hu, Yi Zhou, Qi Zhou

**Affiliations:** 1 Zhiqiang Fu, Department of Thoracic Surgery, Shanghai Shidong Hospital, Shanghai 200438, P.R. China; 2 Liguo Wang, Department of Thoracic Surgery, Shanghai Shidong Hospital, Shanghai 200438, P.R. China; 3 Weigao Hu, Department of Thoracic Surgery, Shanghai Shidong Hospital, Shanghai 200438, P.R. China; 4 Yi Zhou, Department of Thoracic Surgery, Shanghai Shidong Hospital, Shanghai 200438, P.R. China; 5 Qi Zhou, Department of Thoracic Surgery, Shanghai Shidong Hospital, Shanghai 200438, P.R. China

**Keywords:** Early-stage non-small cell lung cancer, Uniportal video-assisted thoracoscopic surgery, Subxiphoid, Intercostal

## Abstract

**Objective:**

To compare the efficacy of subxiphoid and intercostal uniportal video-assisted thoracoscopic surgery (VATS) in patients with early-stage non-small cell lung cancer (NSCLC).

**Methods:**

We performed a retrospective chart review of patients with early-stage NSCLC who underwent subxiphoid uniportal video-assisted thoracic surgery (SA-VATS) or intercostal uniportal VATS (IA-VATS) at Shidong Hospital in Shanghai from November 2020 to May 2023. Perioperative conditions including surgical duration, intraoperative blood loss, postoperative catheterization duration, time to first off-bed activities, and number of lymph node dissected were compared between the groups. Degree of pain, preoperative and postoperative lung function, prognosis, and incidence of complications were also compared between the groups.

**Results:**

Records of a total of 128 patients were included. Of them, 72 patients underwent SA-VATS and 56 patients underwent IA-VATS. The duration of SA-VATS was longer, while the intraoperative blood loss and catheterization times were lower compared to those of IA-VATS (*P*<0.05). Visual analogue scale (VAS) score of patients after SA-VATS was significantly lower than after IA-VATS (*P*<0.05). There was no significant difference in forced expiratory volume in one second (FEV1) between the two groups before the surgery, and one- and 12 months after the surgery (*P*>0.05). The prognosis and the incidence of complications were comparable in the two groups after the surgery (*P*>0.05).

**Conclusions:**

Compared with IA-VATS, SA-VATS is associated with lower intraoperative blood loss, shorter postoperative catheterization and time to first off-bed activities, and less postoperative pain in surgical treatment for early-stage NSCLC. However, there was no significant difference in prognosis and complications between the two approaches.

## INTRODUCTION

Non-small cell lung cancer (NSCLC) accounts for 80%~85% of the total incidence of lung cancer, and is associated with high mortality.^1^,^2^ Video-assisted thoracoscopic surgery (VATS) is the most commonly used minimally invasive surgical method for NSCLC resection, and has the advantages of less trauma, fewer complications, and faster postoperative recovery compared to open chest surgery.^3^,^4^ Uniportal VATS approach requires just a single incision and can further reduce surgery-associated trauma compared to the multiportal VATS. However, traditional intercostal uniportal VATS (IA-VATS) may cause damage to the intercostal nerves, increasing the risk of acute and chronic chest wall pain.^5^,^6^

In 2014, Liu et al.^7^ first reported the subxiphoid uniportal VATS (SA-VATS) lobectomy. It is an innovative modification of VATS. Studies have shown that single subxiphoid incision is effective and prevents excessive injury and compression of the intercostal nerve, and lowers postoperative complications and pain.^8^,^9^ However, this approach is still an invasive treatment, with a certain risk of postoperative complications, including lung leakage, pneumonia, respiratory failure, and atelectasis^7^–^9^ that negatively impact surgical outcomes and postoperative rehabilitation.^9^,^10^

Most studies have compared IA-VATS and SA-VATS for primary lung cancer, pulmonary metastasis, pulmonary benign tumours, and spontaneous pneumothorax.^11^,^12^ However, comparative studies between IA-VATS and SA-VATS for early-stage NSCLC are limited. Thus, the purpose of this study was to compare the effect of intercostal uniportal VATS (IA-VATS) and SA-VATS approaches in surgical resection of early-stage NSCLC.

## METHODS

We performed a retrospective chart review of 128 patients with early-stage NSCLC who underwent uniportal VATS lung resection in Shidong Hospital in Shanghai from November 2020 to May 2023. Based on the medical charts, 72 cases underwent SA-VATS and 56 cases underwent IA-VATS. The decision on the best approach was made according to the discussion of the surgical team.

### Ethical Approval:

It was approved by the ethics committee of our hospital on February 1^st^ 2024, with the number 2024-008-01.

### Inclusion criteria:

- Met NSCLC diagnostic criteria.^13^,^14^
- NSCLC confirmed through pathological examination.
- Age ≥ 18 years old.
- The disease stages I~II.
- Karnofsky Performance Status (KPS) score ≥ 60 points.
- Complete clinical data.
- The lesion is located on one side.

### Exclusion criteria:

- Patients with other malignant tumors.
- Patients with neurological or psychiatric disorders.
- Patients with hematological disorders.
- Patients with systemic or local infections.
- Patients with a history of drug and alcohol dependence.
- Patients with severe internal medicine complications.
- Patients with a history of past pulmonary surgery.

### Operative methods:

The surgical techniques and procedures of IA-VATS and SA-VATS were referred to previous studies.^7^,^15^,^16^

### IA-VATS:

**Fig.1:**
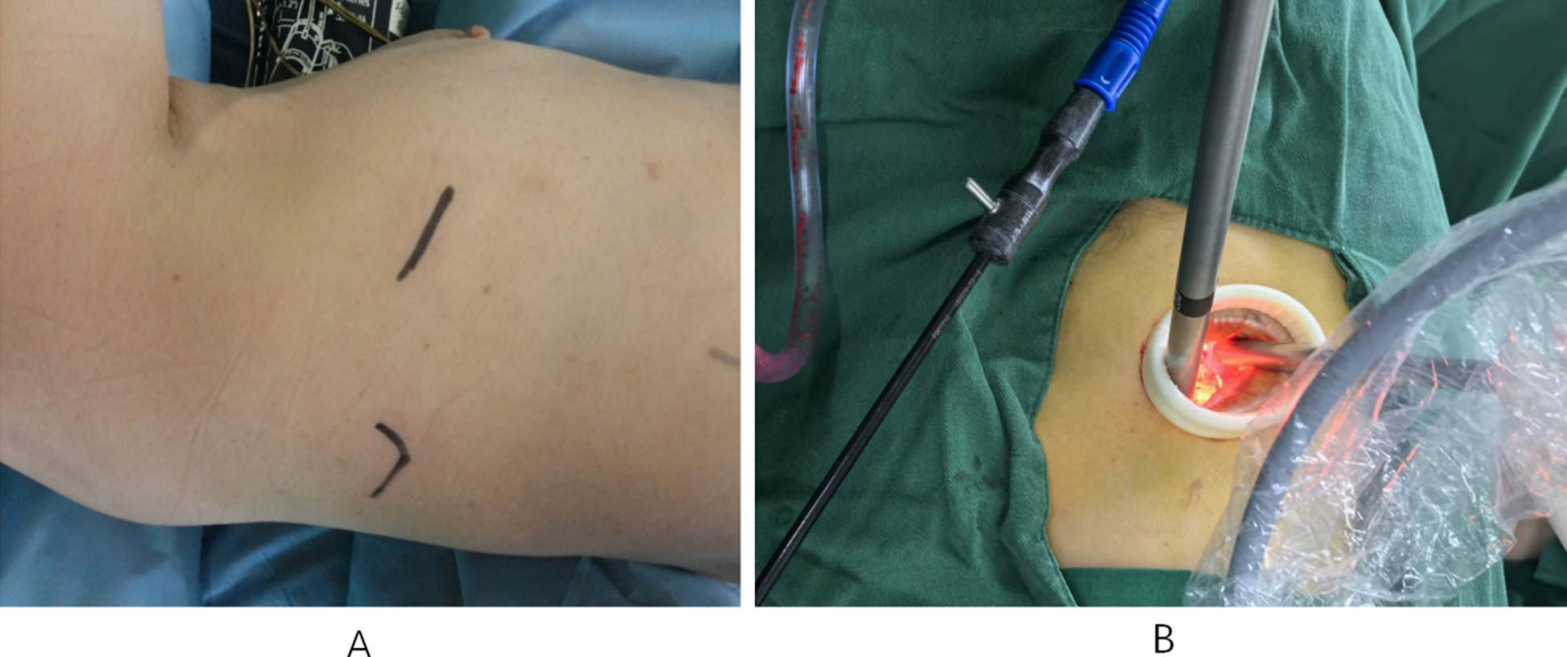
IA-VATS. A. The incision was marked on the skin. B. The thoracoscopic instruments were inserted through the uniport.

Patients were placed in lateral supine position and a single port ventilation was provided on the healthy side. An incision (3-4 cm) was made with the lesion located between the 4th and 5th intercostals at the anterior axillary line as a reference for the manipulation hole. A protective cover was placed at the incision site and a single hole 3D thoracoscopy probe was inserted. Lobectomy was performed with the assistance of endoscopy (Fig.1). The excised specimen was sent for pathological examination. Systematic lymph node dissection, including mediastinal lymph nodes at station three or above and hilar lymph nodes at station 10 was performed. Hemostasis and lung swelling treatment were administered after completing lymph node dissection. Thoracic drainage tube was inserted at the incision site and the incision was closed.

### SA-VATS:

**Fig.2:**
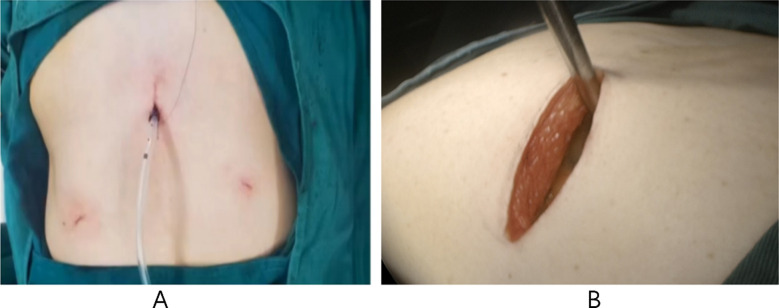
SA-VATS. A. An 4-5cm incision was made of the subxiphoid area. B. Intraoperative process with a hook was inserted through the subxiphoid incision.

Patients were placed in lateral lying position, raising the surgical side trunk by about 30°. An oblique incision (4-5 cm) was made under the xiphoid process to create an operating hole. A protective cover was placed at the incision site, and subcutaneous tissue and rectus abdominis muscle were separated. A subcostal channel was constructed, and mediastinal pleura was opened after reaching the diaphragmatic angle. Condition of the pleura was examined to determine the specific location of the tumor, and lobectomy was performed. (Fig.2) Excised specimen was sent for pathological examination. Lymph node dissection and other procedures were similar to IA-VATS.

### Outcome measures:

Baseline data included gender, age, body mass index (BMI), smoking history, disease staging, lesion diameter, lesion location, and pathological type.

### Perioperative conditions:

Perioperative conditions including surgical duration, intraoperative blood loss, postoperative catheterization duration, time to first off-bed activities, and number of lymph node dissected.

### Pain level:

Pain level of patients was assessed at 2, 12, 24, and 48 hours after the surgery using Visual Analog Scale (VAS). VAS score has a maximum of 10 points, and higher score indicates stronger pain.

### Pulmonary function:

Forced expiratory volume in one second (FEV1) at the first second was measured using a pulmonary function meter before the surgery and 1- and 12 months after the surgery.

Prognosis, including survival rate, distant metastasis rate, and recurrence rate after 12 months of follow-up.

### Complications:

Three months after the surgery, complications such as subendothelial emphysema, arrhythmia, atelectasis, pulmonary leakage, pulmonary infection, pleural effusion were recorded.

### Statistical analysis:

Data analysis was done using SPSS version 26.0 (IBM Corp, Armonk, NY, USA). For continuous variables, results were expressed as mean and standard deviation (SD). Independent sample t-test was used to compare the mean of two independent samples for continuous variables. For categorical variables, frequency distribution was provided and expressed as percentage. Chi square test was used to compare categorical variables between the two groups, such as gender distribution and the presence of smoking. A *p*-value less than 0.05 was considered statistically significant. Repeated measurement analyses were performed when evaluating VAS scores and FEV1 at different time intervals after the surgery. PRISM8.0 software (GraphPad, San Diego, USA) was used for graphics.

## RESULTS

**Fig.3:**
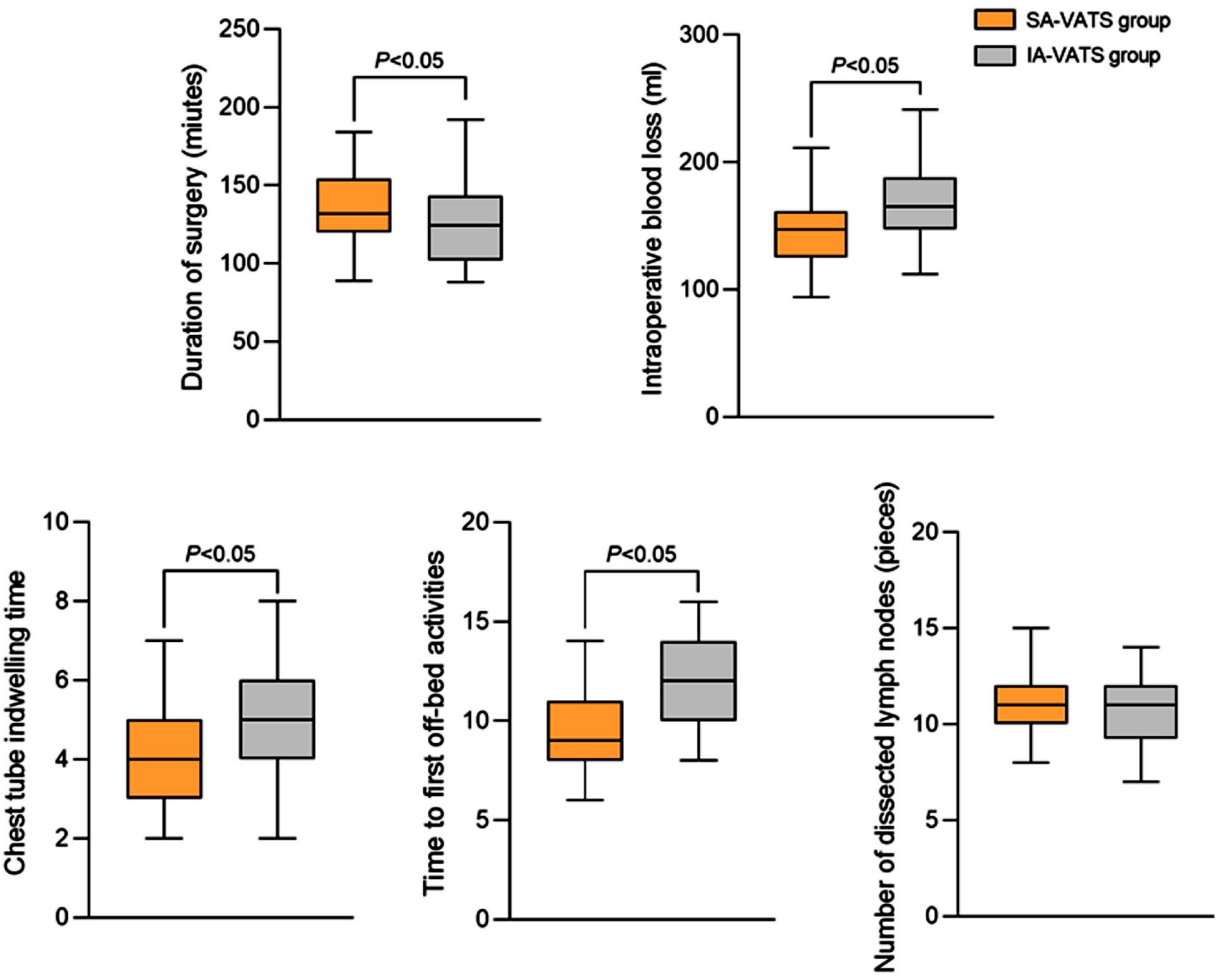
Comparison of perioperative conditions between two groups.

There was no statistically significant difference in baseline data between NSCLC patients who underwent IA-VATS or SA-VATS (*P*>0.05) (Table-I). Fig.3 shows that SA-VATS was associated with significantly longer duration of surgery compared to IA-VATS group. The intraoperative blood loss, postoperative catheterization, and time to first off-bed activities in the SA-VATS group were significantly lower than in the IA-VATS group (*P*<0.05). However, there was no significant difference in the number of dissected lymph nodes between the two groups (*P*>0.05).

**Table-I:** Comparison of baseline data between the groups.

Baseline data	SA-VATS group (n=72)	IA-VATS group (n=56)	t/χ^2^	P
Male (yes)	43 (59.72)	29 (51.79)	0.542	0.462
Age (year)	57.47±7.18	56.45±6.79	0.821	0.413
BMI (kg/m^2^)	22.35±2.83	23.05±2.19	-1.583	0.115
Smoking history (Yes)	40 (55.56)	32 (57.14)	0.032	0.857
Disease staging (I/II)	31/41	28/28	0.611	0.434
Lesion diameter (mm)	21.46±3.96	22.52±4.11	-1.477	0.142
Location of lesion (left/right)	33/39	34/22	2.796	0.094
Pathological type				
Adenocarcinoma	63 (87.50)	47 (83.93)	0.739	0.691
Squamous cell carcinoma	7 (9.72)	8 (14.29)		
Others	2 (2.8)	2 (3.57)		

**Fig.4:**
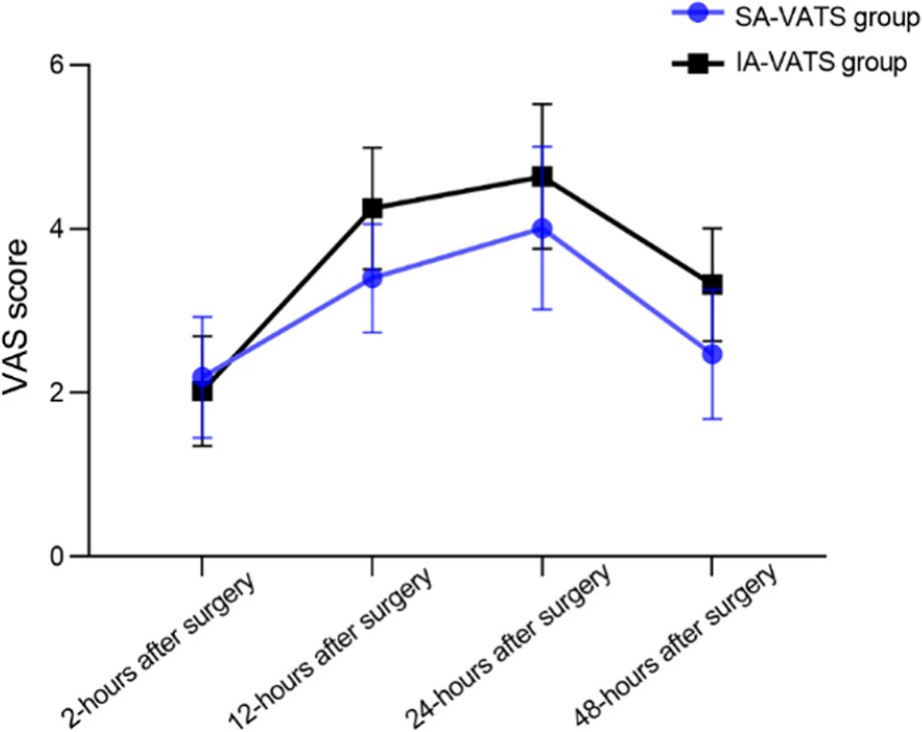
Comparison of postoperative pain between two groups.

**Fig.5:**
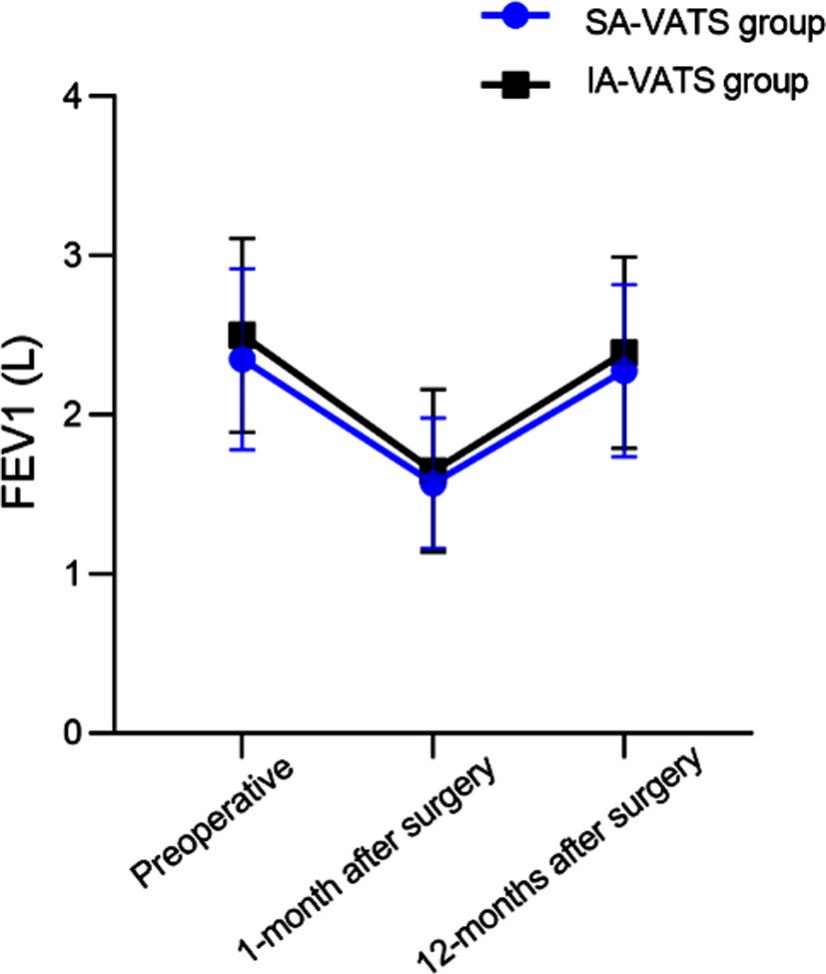
Comparison of lung function.

As demonstrated in Fig.4, pain levels were comparable in both groups two hours after the surgery (*P*>0.05). At 12, 24, and 48 hours after the surgery, VAS scores of the SA-VATS group were significantly lower compared to the IA-VATS group (*P*<0.05). No significant difference in FEV1 levels were detected between the two groups of patients before and after the surgery (Fig.5; *P*>0.05). There was no significant difference in the survival rate, distant metastasis rate, and recurrence rate (Table-II), as well as in the incidence of complications (Table-III) between the SA-VATS and IA-VATS groups (*P*>0.05).

**Table-II:** Comparison of Prognosis between the two Groups.

Group	n	Survival rate	Distant metastasis rate	Recurrence rate
SA-VATS group	72	71 (100.00)	1 (1.39)	1 (1.39)
IA-VATS group	56	54 (98.21)	2 (3.57)	3 (3.57)
*χ^2^*		0.049	0.049	0.590
*P*		0.825	0.825	0.442

**Table-III:** Comparison of complications between the two groups.

Group	n	subcutaneous emphysema	Arrhythmias	Atelectasis of the lungs	Pulmonary leakage	Pulmonary infection	Pleural effusion	Overall incidence rate
SA-VATS group	72	2 (2.78)	1 (1.39)	1 (1.39)	2 (2.78)	1 (1.39)	2 (2.78)	9 (12.50)
IA-VATS group	56	3 (5.36)	0 (0.00)	3 (5.36)	1 (1.79)	2 (3.57)	2 (3.57)	11 (19.64)
*χ^2^*								1.219
*P*								0.270

## DISCUSSION

The results of this study showed that in early NSCLC patients undergoing resection surgery, SA-VATS approach was associated with lower postoperative pain, less intraoperative blood loss, and shorter catheterization duration compared to IA-VATS. There was no significant difference in survival rate, distant metastasis rate, and recurrence rate between two methods. Study by Abdellatef et al.^17^ confirm that the subxiphoid VATS approach can reduce surgical trauma and blood loss, and is associated with relatively mild postoperative pain levels.

Jason Ali et al.^18^ found that uniportal VATS through the xiphoid process can effectively remove lymph nodes, and has short intraoperative hospitalization time of only 9.54 ± 4.67 days. There was no perioperative mortality, and 30-day survival rate of patients reached 100.0%. Studies have pointed out that subxiphoid approach requires relatively little patient positioning, can effectively expose the diseased tissue in the chest cavity, accurately and effectively remove the lesion, and reach the superior sternal fossa directly. That facilitates treatment of thymic veins, avoiding iatrogenic injuries, and shortening the postoperative recovery process.^17^,^18^

A study by Hernandez Arenas LA et al.^19^ also confirmed benefits of SA-VATS approach due to the absence of bony structures around the xiphoid process and no compression of the intercostal nerves. This leads to relatively low levels of postoperative pain which allows patients to start early rehabilitation training. The results of our study are consistent with the above studies. We may speculate that SA_VATS does not require separation of the back muscle group, and therefore does not cause damage to the intercostal nerve and blood vessels. Moreover, it can present the phrenic nerve to the maximum extent during surgery, providing a clear surgical field for the surgent. This, in turn, helps to prevent accidental injury to the bilateral phrenic nerve, and reduces trauma.^17^-^19^

The results of this study show that compared with IA-VATS, SA-VATS is associated with a slightly longer surgical time, but shorter postoperative tube placement and bed movement time, less intraoperative bleeding, and less postoperative pain. It is plausible that during IA-VATS, the instruments need to repeatedly enter and exit the incision, and the angle of the endoscope needs to be constantly adjusted, which can cause compression of the intercostal soft tissue, stimulate and damage intercostal nerves, and cause strong pain.^18^,^19^

SA-VATS can prevent damage to chest wall tissues such as intercostal nerves and muscles, reduce incision pain, facilitate postoperative coughing, sputum excretion, and early activity, and promote postoperative lung recruitment. As this approach requires less stimulation to the chest and abdominal cavity, less bleeding, less postoperative drainage, and shorter catheterization time, it further facilitates early postoperative rehabilitation training.^17^-^19^,^20^ Lee et al.^20^ reported that compared to IA-VATS, SA-VATS thymectomy showed good perioperative outcomes. In addition, SA-VATS has certain advantages in reducing long-term neuropathic pain while causing minimal immediate postoperative pain, supporting the findings of our study.^19^,^20^

However, it is important to note that compared to IA-VATS, SA-VATS is considered more technically-challenging, requires higher surgical skills and more advanced surgical instruments, which may lead to longer surgical duration.^21^ Our study also showed no significant differences in indicators such as lung function, survival rate, distant metastasis rate, and postoperative recurrence rate between the two groups of patients. Our results confirm that SA-VATS surgery not only effectively removes lesions and achieves satisfactory treatment results, but also does not cause lung function damage and has no adverse impact on prognosis. In addition, there was no obvious difference in the incidence of complications between the two groups.

Therefore, both surgical approaches has similar safety, and the complexity of SA-VATS surgery does not increase the risk of complications. It should be noted that the SA-VATS surgical procedure requires the use of specially designed extended double joint instruments that are longer in length, have a certain degree of curvature, can enter the chest cavity at any angle as needed, with reduce interference between the instruments. In addition, during left side surgery, close monitoring of radial artery blood pressure, electrocardiogram, etc. is required to detect signs of any resistance during entry and exit and to prevent damage to the pericardium and heart.

Moreover, prolonged surgery may cause compression of the heart, resulting in reduced bleeding and a decrease in blood pressure that also require continuous monitoring.^21^-^23^ In cases of repeated signs of cardiac compression, adjusting the angle of the operating bed should be considered, and if not effective, intercostal incision should be made to ensure smooth progress of the surgery.^11^,^23^,^24^ A meta-analysis of Mei LX et al.^11^ found that SA-VATS is a safe surgical technique with no significant increase in the incidence of adverse events. In a study of 262 patients, Chen et al.^24^ showed that SA-VATS was associated with significantly lower postoperative pain compared to IA-VATS, but higher incidence of intraoperative arrhythmia. The discrepancy between these results and our observations may be due to the variability in sample sizes or the technical proficiency of the surgical personnel.

### Limitations:

Firstly, this is a retrospective analysis, where all cases were sourced from a single center, which may have possible selection bias. Moreover, mid-term or long-term oncological outcomes of SA-VATS were not explored. Larger multicenter prospective trials with long-term follow-up are needed to verify our results and to assess the long-term effects of both surgical methods.

## CONCLUSION

Compared with IA-VATS, SA-VATS is associated with lower intraoperative blood loss, shorter postoperative catheterization time and time to first off-bed activities, and less postoperative pain in surgical treatment for early-stage NSCLC. However, there is no significant difference in prognosis and complications between the two approaches.

### Authors’ Contributions:

**ZF:** Concept, study design, literature search and manuscript writing.

**LW, WH** and **YZ:** Data collection, data analysis, interpretation and critical review.

**QZ:** Revision of manuscript and validation.

All authors have read, approved the final manuscript and are responsible for the integrity of the study.

## References

[1] Abu Rous F, Singhi EK, Sridhar A, Faisal MS, Desai A (2023). Lung Cancer Treatment Advances in 2022. Cancer Invest.

[2] Cihanbeylerden M, Yumrukuz M, Kurt B, Tuccar C, Safak C (2022). Effect of Pulmonary Functions on Survival in Patients with Operable Non-small Cell Lung Cancer. J Coll Physicians Surg Pak.

[3] Oda R, Okuda K, Osaga S, Watanabe T, Sakane T, Tatematsu T (2019). Long-term outcomes of video-assisted thoracoscopic surgery lobectomy vs. thoracotomy lobectomy for stage IA non-small cell lung cancer. Surg Today.

[4] Fujita T, Koyanagi A, Kishimoto K (2024). Complete thoracoscopic lobectomy versus hybrid video-assisted thoracoscopic lobectomy for non-small cell lung cancer. Gen Thorac Cardiovasc Surg.

[5] Qiu Y, Zhou J, Wu D, Luo A, Yang M, Zheng Q (2024). Suction versus non-suction drainage strategy after uniportal thoracoscopic lung surgery:a prospective cohort study. J Thorac Dis.

[6] Tulinský L, Kepičová M, Ihnát P, Tomášková H, Mitták M, Staníková L (2023). Radicality and safety of mediastinal lymphadenectomy in lung resection:a comparative analysis of uniportal thoracoscopic, multiportal thoracoscopic, and thoracotomy approaches. Surg Endosc.

[7] Liu CC, Wang BY, Shih CS, Liu YH (2014). Subxiphoid single-incision thoracoscopic left upper lobectomy. J Thorac Cardiovasc Surg.

[8] Hernandez-Arenas LA, Lin L, Yang Y, Liu M, Guido W, Gonzalez-Rivas D (2016). Initial experience in uniportal subxiphoid video-assisted thoracoscopic surgery for major lung resections. Eur J Cardiothorac Surg.

[9] He G, Yao T, Zhao L, Geng H, Ji Q, Zuo K (2024). A proof-of-concept study:advantages of the subxiphoid over the lateral intercostal approach. Interdiscip Cardiovasc Thorac Surg.

[10] Hong Z, Sheng Y, Bai X, Cui B, Lu Y, Wu X (2023). Clinical efficacy of robot-assisted subxiphoid versus lateral thoracic approach in the treatment of anterior mediastinal tumors. World J Surg Oncol.

[11] Mei LX, Wang YY, Chen Y, Dai L, Chen MW (2022). Subxiphoid versus intercostal video-assisted thoracic surgery for lung resection:a meta-analysis. Minim Invasive Ther Allied Technol.

[12] Li L, Tian H, Yue W, Li S, Gao C, Si L (2016). Subxiphoid vs intercostal single-incision video-assisted thoracoscopic surgery for spontaneous pneumothorax:A randomised controlled trial. Int J Surg.

[13] Health Commission Of The People's Republic Of China N (2022). National guidelines for diagnosis and treatment of lung cancer 2022 in China (English version). Chin J Cancer Res.

[14] Oncology Society of Chinese Medical Association;Chinese Medical Association Publishing House (2023). [Chinese Medical Association guideline for clinical diagnosis and treatment of lung cancer (2023 edition). Zhonghua Zhong Liu Za Zhi.

[15] Liu YW, Chou SH, Chou A, Kao CN (2022). Simultaneous Comparison of Subxiphoid and Intercostal Wound Pain in the Same Patients Following Thoracoscopic Surgery. J Clin Med.

[16] Cai H, Xie D, Al Sawalhi S, Jiang L, Zhu Y, Jiang G (2020). Subxiphoid versus intercostal uniportal video-assisted thoracoscopic surgery for bilateral lung resections:a single-institution experience. Eur J Cardiothorac Surg.

[17] Abdellateef A, Ma X, Chen Z, Wu L, Cai J, Jiang L (2020). Subxiphoid uniportal thoracoscopic pulmonary segmentectomy for stage I non-small cell lung cancer:Feasibility, quality of life and financial worthiness. Thorac Cancer.

[18] Ali J, Haiyang F, Aresu G, Chenlu Y, Gening J, Gonzalez-Rivas D (2018). Uniportal Subxiphoid Video-Assisted Thoracoscopic Anatomical Segmentectomy:Technique and Results. Ann Thorac Surg.

[19] Hernandez-Arenas LA, Guido W, Jiang L (2016). Learning curve and subxiphoid lung resections most common technical issues. J Vis Surg.

[20] Lee J, Cho S, Yoon SH, Shih BCH, Jung W, Jeon JH (2023). Surgical outcomes of thoracoscopic thymectomy via the single-port subxiphoid approach versus the unilateral intercostal approach. Interdiscip Cardiovasc Thorac Surg.

[21] Elkhayat H, Hamza HM, Elshoieby MH, Omar MI, Gaber EA (2022). Role of subxiphoid uniportal video-assisted thoracoscopic surgery in pulmonary metastasectomy. Kardiochir Torakochirurgia Pol.

[22] Wang J, Xu M, Zhang C, Wei D (2022). Clinical analysis of subxiphoid single-port thoracoscopic surgery for simultaneous bilateral lung lesion resection. BMC Surg.

[23] Suda T (2017). Subxiphoid Uniportal Video-Assisted Thoracoscopic Surgery Procedure. Thorac Surg Clin.

[24] Chen Z, Jiang L, Zheng H, Zhang W, Lv X, Abdellateef A (2022). Early postoperative pain after subxiphoid uniportal thoracoscopic major lung resection:a prospective, single- blinded, randomized controlled trial. Interact Cardiovasc Thorac Surg.

